# Juvenile idiopathic arthritis-associated RIG-I S144F variant inhibits type I interferon signaling by blocking TRIM25-mediated ubiquitination

**DOI:** 10.1016/j.gendis.2025.101791

**Published:** 2025-08-07

**Authors:** Li Zhang, Song Zhang, Yaoyao Shangguan, Wanming Huang, Haolan Jin, Lili Li, Yuyin Qi, Yuxia Zhang, Ping Zeng, Huifang Xian

**Affiliations:** a Guangzhou Institute of Pediatrics, Guangzhou Women and Children’s Medical Center, Guangzhou Medical University, Guangzhou, Guangdong 510623, China; b The Second Affiliated Hospital of Wenzhou Medical University, Wenzhou, Guangdong 325000, China; c Department of Allergy, Immunology and Rheumatology, Guangzhou Women and Children’s Medical Center, Guangzhou Medical University, Guangzhou, Guangdong 510623, China

Juvenile idiopathic arthritis (JIA) is a heterogeneous group of idiopathic inflammatory arthritis affecting children before 16 years of age.^1^ Although the pathogenesis of JIA remains unclear, viral infection is a potential environmental risk factor in JIA development.^2^ The retinoic acid-inducible gene I (RIG-I), a key cytosolic pathogen recognition receptor (PRR) responsible for detecting viral double-stranded RNA (dsRNA), serves as a critical gatekeeper of antiviral immunity through type I interferon (IFN) induction.^3^ Recent study suggests that the S144F variant is associated with increased disease severity and poorer treatment outcomes in pediatric rheumatic patients.^4^ However, the potential contribution and mechanism of RIG-I genetic variants to JIA susceptibility has never been systematically explored. In this study, we show that the RIG-I S144F variant is significantly enriched in JIA patients. S144F variant reduces its binding with the E3 ligase TRIM25, leading to decreased K63-linked polyubiquitination of RIG-I. Consequently, this results in impaired type I interferon signaling activation, higher viral load, and aggravated NF-κB signaling. In summary, we uncover a critical role of the RIG-I S144F variant in suppressing antiviral immunity, and shed light on immune pathogenesis of JIA.

To explore the relationship between viral infection and JIA development, we examined the mutation frequency of viral nucleic acid sensors by whole exome sequencing (WES). Twenty-three JIA patients under 16 years old diagnosed according to International League of Associations for Rheumatology (ILAR) criteria formed our study cohort (Fig. 1A). We found *CGAS*, *RIGI*, *IFIH1* and *TLR3* variants in WES results of patients (Fig. 1B; Table S1). Markedly, RIG-I shown high mutation frequency compared with the East Asian population from 1000 Genome Project (Fig. 1B). We observed three variants of RIG-I, including M51V (4%), S144F (26%) and D580E (9%) in JIA cohort (Fig. 1C). Notably, the mutation frequency of RIG-I S144F in JIA was strikingly increased compared with the mutation frequency of East Asian population from 1000 Genome Project which is about 4% (Table S1). We further enrolled samples for single nucleotide polymorphism (SNP) analysis. We accessed S144F (rs55789327) in 74 healthy donors and 79 JIA patients, including 21 systemic-onset JIA (so-JIA) patients. SNP genotyping result suggested that S144F variant shows enrichment in JIA patients (16.45%), especially in so-JIA patients (23.80%), compared with healthy donors (12.16%) (Fig. 1D). Thus, our results indicate that RIG-I may function as a risk gene for JIA.

**Figure 1:**
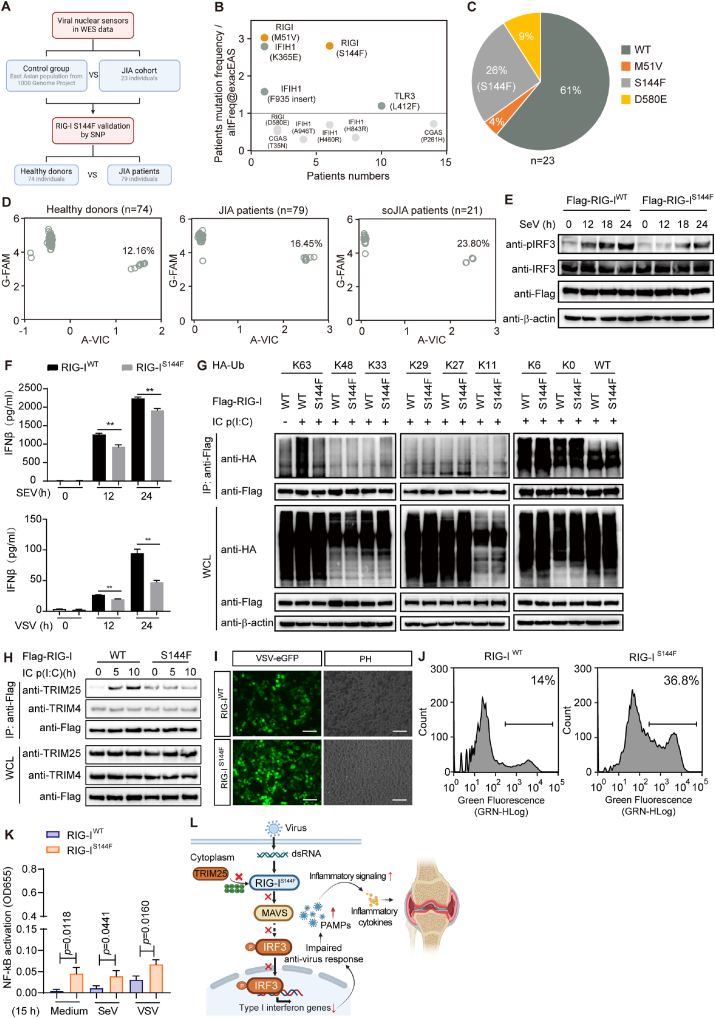
S144F variant of RIG-I identified from JIA patients impairs type I interferon signaling, leading to the increased viral load and hyperactivation of NF-κB signaling. **(A)** Schematic for patient enrollment. **(B)** Diagram presents variants of viral nucleic acid sensors identified from our JIA cohort. *Y*-axis shows the ratio of mutation frequency (JIA cohort:East Asian population from 1000 Genome Project). **(C)** Mutation frequency of RIG-I variants in JIA cohort. **(D)** SNP validation of S144 in healthy donors (*n* = 74) and JIA patients (*n* = 79). **(E)** Immunoblot analysis of extracts of RIG-I^WT^ or RIG-I^S144F^ reconstructed A549 cells infected with SeV for the indicated time points. **(F)** ELISA analysis of IFN-β in the supernatant of RIG-I^WT^ or RIG-I^S144F^ reconstructed A549 cells with SeV infection. **(G)** HEK 293T cells were transfected with Flag-RIG-I^WT^ or Flag-RIG-I^S144F^, together with different types of HA-Ub. Lysates were collected for immunoblot and co-immunoprecipitation. **(H)** Co-immunoprecipitation analysis of HEK293T cells transfected with Flag-RIG-I^WT^ or Flag-RIG-I^S144F^, followed by poly(I:C) stimulation. **(I)** Phase-contrast (PH) and fluorescence microscopy analyses in RIG-I^WT^ or RIG-I^S144F^ reconstructed A549 cells infected with VSV-eGFP for 18 h. Scale bars, 100 μm. **(J)** Flow cytometric analyses of RIG-I^WT^ or RIG-I^S144F^ reconstructed A549 cells infected with VSV-eGFP. **(K)** SEAP activity in NF-κB reporter HEK 293T cells transfected with RIG-I^WT^ or RIG-I^S144F^, followed by infection with SeV and VSV. **(L)** Illustration of RIG-I S144F variant in antiviral immune response and JIA development. Data are means ± SD of 3 independent experiments. ∗*P* < 0.05, ∗∗*P* < 0.01 and ∗∗∗*P* < 0.001.

Two caspase-activation and recruitment domains (2CARDs) on N-terminal is known as the functional domain of RIG-I. Therefore, we focused on the function of M51V and S144F variants in IFN-I signaling. We rescued M51V and S144F variants in RIG-I KO 293T cells and detected IFN-β transcriptional activity. S144F mutation significantly reduced RIG-I-mediated IFN-I signaling, but not M51V (Fig. S1A and S1B). To further verify the function of S144F in IFN-I signaling, we rescued RIG-I WT or S144F in RIG-I-KO A549 cells respectively (Fig. S2A and S2B). Consistently, RIG-I^S144F^ reconstitution attenuated IRF3 activation (Fig. 1E), interferon stimulated genes (ISGs) transcription (Fig. S2C and S2D) and IFN-β expression (Fig. 1F) compared with WT upon SeV or VSV infection. Therefore, S144F variant impaired RIG-I-induced IFN-I signaling.

To find out the mechanism of S144F variant in IFN-I signaling, we next assessed the protein stability of RIG-I WT and S144F. Cycloheximide (CHX) was applied to inhibit protein synthesis in eukaryotes. We found that S144F variant show no influence on RIG-I protein degradation (Fig. S3A and S3B). Given that the activity of RIG-I is tightly regulated by multiple ubiquitination,^5^ we explored the ubiquitination of RIG-I WT and variant upon stimulation. By co-immunoprecipitation, we found that S144F variant significantly inhibited the K63-linked ubiquitination modification of RIG-I but not other ubiquitin chains (Fig. 1G). TRIM25 and TRIM4 are E3 ligases for K63-linked polyubiquitination modification in RIG-I activation upon RNA viral infection. We observed increased interaction between RIG-I WT with endogenous TRIM25, but not TRIM4, upon intracellular poly(I:C) (IC p(I:C)) treatment. Meanwhile, S144F variant decreased RIG-I-TRIM25 binding upon stimulation (Fig. 1H). Taken together, these results demonstrate that S144F suppresses RIG-I-induced antiviral innate immune responses by impairing TRIM25-mediated K63-linked polyubiquitination of RIG-I.

IFN-I signaling is important in antiviral immune response. Given that S144F restrained RIG-I-induced IFN-I signaling, we wondered if S144F variant might also regulate inflammatory signaling upon viral infection. A549 cells with stable expression of RIG-I S144F variant showed increased viral load in response to eGFP-VSV infection, compared with RIG-I WT (Fig. 1I and J; Fig. S4). Furthermore, we observed striking NF-κB hyperactivation in RIG-I S144F rescued NF-κB reporter cells in contrast with WT (Fig. 1K).

In conclusion, our results demonstrated that S144F variant blocked K63-linked ubiquitination of RIG-I upon viral infection. The attenuated IFN-I signaling by RIG-I S144F variant leads to the overload of virus. Virus infection provides attendant pathogen-associated molecular patterns (PARPs), which can be recognized by various pattern recognition receptors (PRRs) and initiated innate immune response, including NF-κB signaling and inflammasome activation. Thus, the overload of virus maybe responsible for the excessive inflammatory signaling. This study illustrates the mechanism of attenuated IFN-I signaling mediated by RIG-I S144F variant, and reveals the relevance of defective anti-viral immunity and JIA development.

## CRediT authorship contribution statement

**Li Zhang:** Investigation, Data curation, Project administration, Writing – original draft. **Song Zhang:** Data curation, Validation. **Yaoyao Shangguan:** Investigation, Resources. **Wanming Huang:** Project administration, Data curation. **Haolan Jin:** Investigation. **Lili Li:** Validation. **Yuyin Qi:** Data curation. **Yuxia Zhang:** Investigation. **Ping Zeng:** Writing – review & editing. **Huifang Xian:** Writing – review & editing, Data curation, Supervision.

## Ethics declaration

We recruited cases and controls from Guangzhou Women and Children’s Medical Center, Guangzhou Medical University. The study was approved by the Medical Ethics Committees of the recruiting hospital (2016111853).

## Data availability

All data needed to evaluate the conclusion in the paper are presented in the manuscript, Supplementary Material and Supplementary Table 1. Further inquiries can be directed to the corresponding authors.

## Funding

This study was supported by funding from the 10.13039/501100001809National Natural Science Foundation of China (No. 32100735), 10.13039/501100010008China Postdoctoral Science Foundation (No. 2019M663225), Guangdong Provincial Basic and Applied Basic Research Foundation (China) (No. 2025A1515012735), and Guangzhou Municipal Science and Technology Project (China) (No. 2024A03J0806).

## Conflict of interests

The authors declare that they have no known competing financial interests or personal relationships that could have appeared to influence the work reported in this paper.
