# Supplementary material for: Juvenile idiopathic arthritis-associated RIG-I S144F variant inhibits type I interferon signaling by blocking TRIM25-mediated ubiquitination
Source: Genes Dis. 2025 Aug 7;13(4):101791. doi: 10.1016/j.gendis.2025.101791 (PMC13011034; doi:10.1016/j.gendis.2025.101791)
Supplement: Multimedia component 1 [file mmc1.docx]

**Methods and supplementary figures**

Title: Juvenile idiopathic arthritis-associated RIG-I S144F Variant Inhibits Type I Interferon Signaling by Blocking TRIM25-mediated Ubiquitination

**Materials and Methods**

**Patients**

We recruited cases and controls from Guangzhou Women and Children’s Medical Center, Guangzhou Medical University. The study was approved by the Medical Ethics Committees of the recruiting hospital (2016111853). Informed written consent was obtained from the legal guardians of all participants. The study was carried out from October 2016 to April 2019 using samples of children (n_Healthy donors_ = 74, n_patients_ = 102) who attended the Immunology Department in a tertiary pediatric hospital in Guangzhou, China.

**Cell culture and transfection**

HEK293T and A549 cells were maintained in Dulbecco's modified Eagle's medium (Gibco, C11995500BT) supplemented with 10% fetal bovine serum (Gibco, 10099-141) at 37°C in 5% CO_2_. Overexpression plasmids were transfected into the cells using Lipofectamine 3000 reagent (Invitrogen, L3000015) according to the manufacturer's instructions.

**Antibodies and reagents**

Monoclonal anti-Flag M2-peroxidase (A8592), monoclonal anti-β-actin antibody produced in mouse AC-74 (A2228), anti-Flag M2 affinity gel (A2220), and Cycloheximide (C1988) were purchased from Sigma. Anti-HA-peroxidase (high affinity from rat immunoglobulin G1) (12013819001) were purchased from Roche and TRIM4 (ab229952) was purchased from Abcam. RIG-I (D14G6) rabbit monoclonal antibody (mAb) (3743S), phospho-IRF3 (Ser396, 4D4G) rabbit mAb (37829S), IRF3 (D6I4C) rabbit mAb (11904S), and TRIM25 (D9T7G) rabbit mAb (13773) were purchased from Cell Signaling Technology. Poly(I:C)-LMW was purchased from Invivogen (tell-pic).

**Luciferase Reporter Assays**

Cells were seeded in 24-well plates and transfected with indicated DNA plasmids, including plasmids encoding IFN-β luciferase reporter gene (firefly luciferase; 20ng) and pRL-TK (*Renilla* luciferase plasmid; 10ng), together with indicated plasmids (100 ng). After stimulation with SeV, the cells were lysed using a passive lysis buffer from Promega. Enzyme activity was normalized by the efficiency of transfection on the basis of *Renilla* luciferase activity levels. Fold induction relative to the basal level was measured. The values were means ± SD of three independent transfections performed in parallel.

**Immunoprecipitation and immunoblot analysis**

Protein was extracted in an ice-cold low-salt lysis buffer containing 50 mM Hepes (pH 7.5), 150 mM NaCl, 1 mM EDTA, 1.5 mM MgCl_2_, 10% glycerol, and 1% Triton X-100, along with a protease inhibitor cocktail (5 mg/ml; Roche, 5892791001). The immunoprecipitation experiments were carried out by incubating the whole-cell extracts overnight with anti-Flag agarose gels (Sigma, A2220), then washing the beads three times with the low-salt lysis buffer. Collections were boiled for 5 minutes in 2× SDS loading buffer (FD Biotechnology，FD006). The samples were electrophoresed on SDS-polyacrylamide gels ranging from 8% to 12% and transferred onto polyvinylidene fluoride membranes, which were then blocked using 5% skim milk (BioRad, 1706404). The membranes were then subjected to antibody incubation and detection using enhanced chemiluminescence (Millipore, WBKLS0500).

**KO of RIG-I by the CRISPR-Cas9 system and rescued cell line generation**

We analyzed guide RNA (gRNA) in the website http://crispr.mit.edu/ and chose the gRNA sequence with the highest score to design primers: RIG-I guide, 5′-AGATCAGAAATGATATCGGT-3′. Annealing products were annealed and then linked to the pCRISPR-V2 vector. For the generation of rescued cell lines, stable overexpression plasmids of RIG-I WT and RIG-I S144F mutants were conducted the synonymous mutation at the single-guide RNA targeting sequence.

**RNA extraction, RNA sequencing, and real-time PCR analysis**

After total cellular RNA was extracted by Trizol reagent (Invitrogen, 15596018CN), reverse transcription was performed by a reverse transcription kit (Vazyme, R223-01). Real-time PCR was conducted using SYBR Green qPCR Mix (Vazyme, Q711-02). The data were normalized to the GAPDH gene and analyzed using Ct models to calculate the relative abundance of transcripts. Table 1 in Supplementary Material shows the list of primers for real-time PCR.

**Enzyme-linked immunosorbent assay (ELISA).**

The concentration of human IFN-β protein in cell culture supernatant was estimated with ELISA kits (InvivoGen, luex-hifnbv2) according to the manufacturer’s recommendations.

**NF-κB reporter assay**

NF-κB reporter HEK 293T cells stably express the human NOD2 gene and an NF-κB-inducible SEAP reporter gene (InvivoGen, hkb-hnod2v2). Cells were seeded in 96-well plates and transfected with indicated DNA plasmids. After transfection for 12 h, cell culture medium was replaced with QUANTI-Blue™ detection medium (InvivoGen, rep-qbs), a SEAP detection reagent, together with SeV or VSV stimulation. Plates were incubated at 37 °C in 5% CO_2_ for 15 h. SEAP can be determined by spectrophotometer at 620-655 nm.

**SNP Genotyping**

Genomic DNA extraction kit (TIANGEN, DP304) was used to prepare genomic DNA from blood according to the manufacturer's protocol. The samples were genotyped using a TaqMan platform from Applied Biosystems in Foster City, CA. To guarantee precision, a blinded method was utilized in the genotyping procedure. Eight negative and positive control samples were included in each 384-well plate to monitor quality control. Genotyping sequence [VIC/FAM] is as followed, TGCACCTGCCATCATCCCCTTAGTA[A/G]AGCAAATCTAAGCAAGGTAACTGTA.

**Statistical analysis**

Student’s t-test was used for functional luciferase statistical analyses with GraphPad Prism 7.0 software. * P < 0.05, ** P < 0.01, *** P < 0.001.


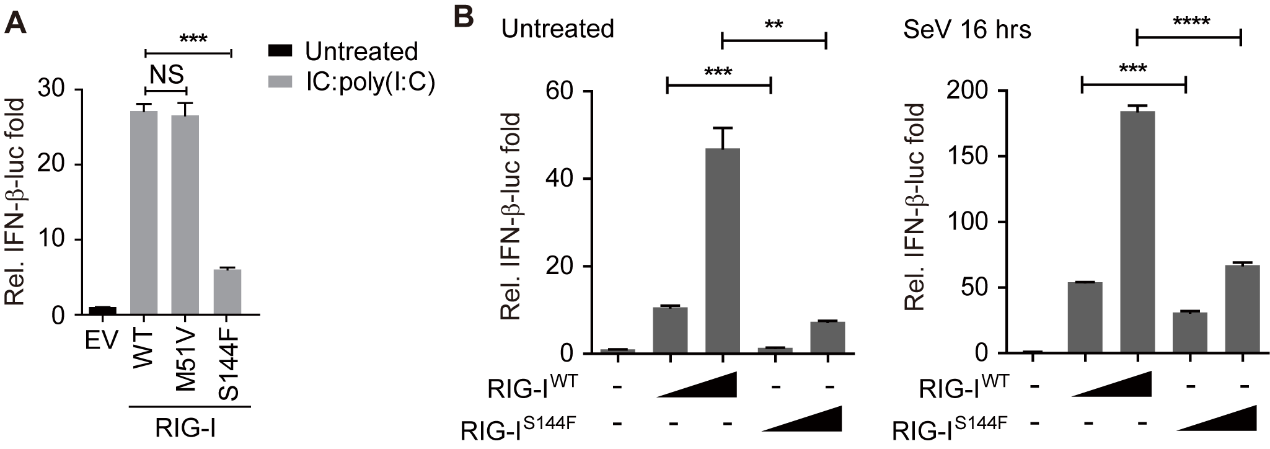


**Figure S1. S144F mutation impairs RIG-I induced type I interferon signaling activation.** (**A**) Luciferase activity in RIG-I KO 293T cells transfected with empty vector (EV), RIG-I-WT, RIG-I-M51V or RIG-I-S144F, together with an IFN-β luciferase (IFN-β-luc) reporter, followed by treatment with IC poly(I:C). (**B**) Luciferase activity in RIG-I KO 293T cells transfected with EV, increasing amounts of expression vector for RIG-I-WT or RIG-I-S144F, together with an IFN-β-luc reporter, with or without SeV infection.


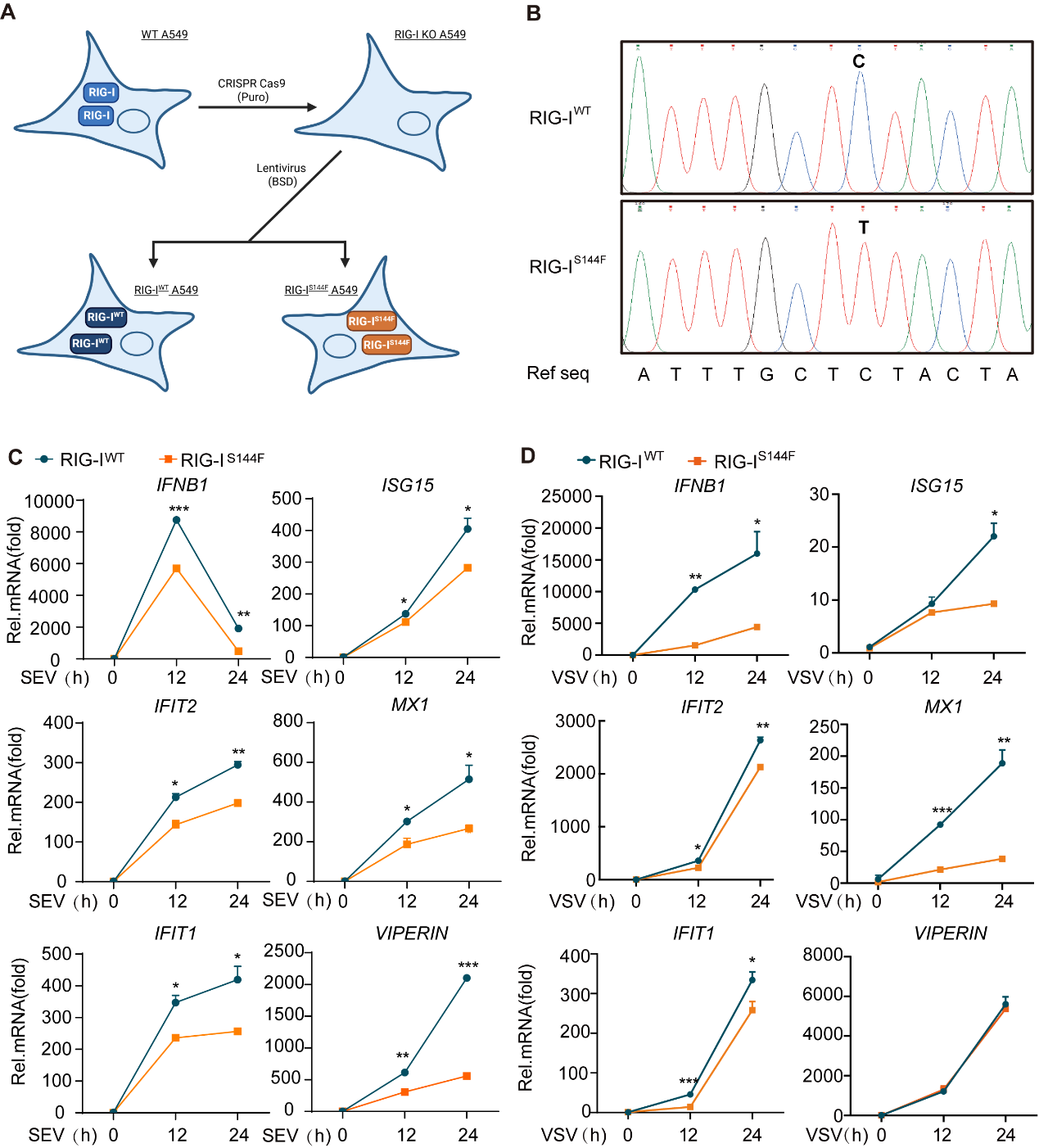


**Figure S2. S144F suppresses RIG-I-induced antiviral immune response.** (**A**) Schematic of RIG-I^WT^ and RIG-I^S144F^ A549 cells construction. (**B**) Sequencing results of RIG-I^WT^ and RIG-I^S144F^ reconstructed A549 cells. (**C** and **D**) Real-time PCR analysis of IFNB1 and ISGs in RIG-I^WT^ and RIG-I^S144F^ reconstructed A549 cells after SeV (C) or VSV (D) infection.


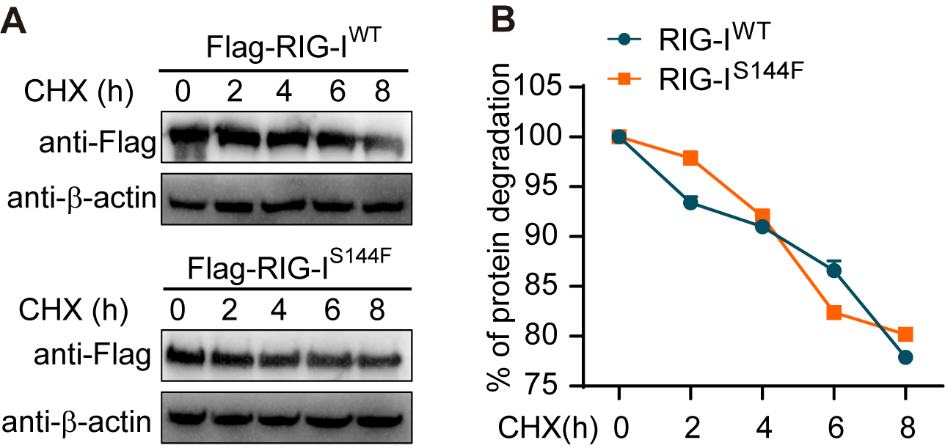


**Figure S3. Protein degradation rate of RIG-I^WT^ and RIG-I^S144F^.** (**A**) HEK293T cells transfected with Flag-RIG-I^WT^ or Flag-RIG-I^S144F^ were treated with Cycloheximide (CHX, 100 μg/mL) for indicated time course. Lysates were collected for immunoblot. (**B**) Quantification result of protein degradation rate.


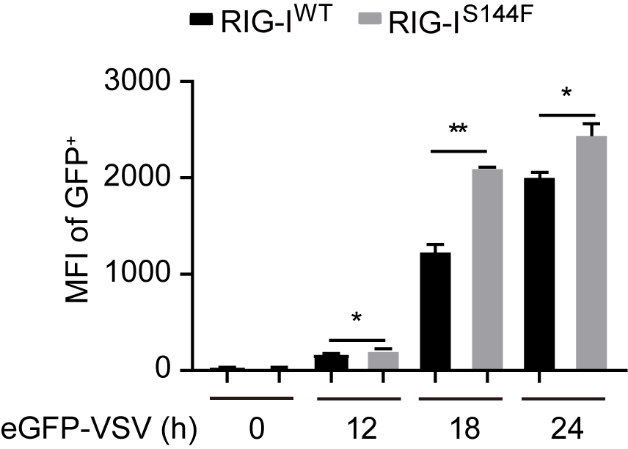


**Figure S4. S144F mutation leads to increased viral load.** Flow cytometric analyses of RIG-I^WT^ or RIG-I^S144F^ reconstructed A549 cells infected with VSV-eGFP for indicated time point.

**Table1. The following primers were used for real-time PCR:**

| **Genes** |  | **Sequence(5'to3')** |
| --- | --- | --- |
| *RPL13A* | Foward | GCCATCGTGGCTAAACAGGTA |
|  | Reverse | GTTGGTGTTCATCCGCTTGC |
| *IFNβ* | Foward | GATGAACTTTGACATCCCTGAG |
|  | Reverse | TCAACAATAGTCTCATTCCAGC |
| *ISG15* | Foward | CGCAGATCACCCAGAAGATCG |
|  | Reverse | TTCGTCGCATTTGTCCACCA |
| *IFIT1* | Foward | TCAGGTCAAGGATAGTCTGGAG |
|  | Reverse | AGGTTGTGTATTCCCACACTGTA |
| *IFIT2* | Foward | TATTGGTGGCAGAAGAGGAAGA |
|  | Reverse | CAGGTGAAATGGCATTTTAGTT |
| *VIPERIN* | Foward | TGGGTGCTTACACCTGCTG |
|  | Reverse | GAAGTGATAGTTGACGCTGGTT |
| *MX1* | Foward | GTTTCCGAAGTGGACATCGCA |
|  | Reverse | CTGCACAGGTTGTTCTCAGC |
